# A Proof of Concept Study Demonstrating the Feasibility of a Text Message Prompt for Hepatitis C Testing in the 1945-1965 Birth Cohort

**DOI:** 10.7759/cureus.4745

**Published:** 2019-05-24

**Authors:** Rebecca J Fisk, Disha Kumar, John B Kellogg, Daniel R Murphy, Kristen A Staggers, Monisha Arya

**Affiliations:** 1 Epidemiology and Public Health, University of Texas Health Science Center at Houston School of Public Health, Houston, USA; 2 Miscellaneous, Baylor College of Medicine, Houston, USA; 3 Internal Medicine, Baylor College of Medicine, Houston, USA; 4 Neurosurgery, Baylor College of Medicine, Houston, USA

**Keywords:** hepatitis c, patient education, screening, hepatitis c virus, testing

## Abstract

Purpose

Despite national recommendations stating all individuals in the 1945-1965 “birth cohort” be tested for hepatitis C virus (HCV), testing rates remain low. The purpose of this proof of concept study was to assess the feasibility of text messaging to promote HCV testing among birth cohort patients.

Methods

Participants were assigned to receive a text message to promote HCV testing, or a general health message as a control. Participants were sent the message immediately prior to an upcoming appointment. Patients not enrolled in the study were in the standard-of-care group. To assess the impact of the text on HCV test orders on the appointment date participant charts were reviewed.

Results

The sample was largely non-Hispanic, Caucasian, and female. Of participants sent the HCV message (n = 22), 50.0% had a test ordered, compared to 41.7% and 27.5% in the control (n = 13) and standard-of-care groups (n = 69), respectively.

Conclusion

This proof of concept study demonstrated the feasibility of text messaging to promote HCV testing among birth cohort patients. Those receiving the HCV message were more likely to have an HCV test ordered compared to those who received no message, although this difference was not statistically significant. A larger study is needed to confirm these results.

## Introduction

Over 50% of the 3.2 million Americans living with hepatitis C virus (HCV) do not know they are infected, because they have not been tested [1,2]. To combat this epidemic, the US Centers for Disease Control and Prevention (CDC) recommends that all individuals in the 1945-1965 birth cohort be tested for HCV once [2]. However, testing rates remain low in this population due in part to physicians’ time limitations during visits, discomfort with the subject of HCV, and lack of awareness of testing recommendations [3,4]. To overcome physician barriers, we designed a text message campaign to educate and activate patients to engage their physicians about HCV testing. Text messaging has been effective in prompting patients to engage in their health, but has not been used to improve HCV testing among birth cohort patients [5]. The objective of this proof of concept study was to assess the feasibility of a text messaging campaign to increase HCV testing in birth cohort patients in primary care.

## Materials and methods

For this proof of concept study, we recruited patients between June-August 2017 and March-April 2018 at an internal medicine clinic in Houston, Texas. With assistance from a site champion, we identified patients with an upcoming preventative care appointment and recruited them by phone. Patients were eligible if they planned to attend the appointment, had a cell phone capable of receiving text messages, and spoke English. This study was approved by the Baylor College of Medicine Institutional Review Board.

After obtaining consent for participation, we assigned participants in the birth cohort to receive one of two educational messages about HCV or a general health message (e.g., dietary information) as an attention control. One HCV testing message focused on the national recommendation for testing adults in the 1945-1965 birth cohort, and the other reviewed the asymptomatic nature of chronic HCV infections. In order to keep the number of participants in each text message arm equal for this proof of concept study, as each participant enrolled in the study, we sequentially assigned them to receive one of the three text messages (e.g., HCV 1, HCV 2, control, HCV 1, HCV 2, control). All text messages included the participant’s name, the name of the physician they were seeing that day, and a cue to ask their physician about the topic of the health message. The text message was sent one hour before each patient’s appointment.

After all text messages were sent, we reviewed the electronic medical record (EMR) to see if an HCV test was ordered on the appointment date - the primary outcome of the study. Information on demographics and HCV tests completed prior to the appointment date (i.e., before the research study began) was also collected during the EMR review. Patients who missed or cancelled their appointments were excluded. To estimate the clinic’s baseline HCV testing rate, we analyzed HCV test orders for birth cohort patients with upcoming visits, but not contacted by the research staff (“background population”). For this proof of concept study, descriptive statistics and crude odds ratios were calculated using Stata version 15.1 (StatCorp LLC, College Station, Texas).

## Results

In this proof of concept study, we enrolled 35 birth cohort participants. One birth cohort participant was inadvertently not sent the text message and was therefore excluded in the analysis. The final analytic sample was 34 birth cohort participants, 22 of whom were sent an HCV message. The background population contained 69 birth cohort patients, and was used to estimate background testing rates. This group was made up of birth cohort patients with upcoming preventive care appointments, but not contacted by the research team. Overall, the sample was largely non-Hispanic, Caucasian, and female (Table 1). Among all birth cohort patients, 27.2% had a previous HCV test.

**Table 1:** 1945-1965 Birth Cohort Patient Demographics *One patient in the Hepatitis C message group did not have ethnicity information recorded in their EMR. HCV: Hepatitis C virus; EMR: Electronic medical record.

		Background Population (n = 69)	General Health Message Group (n = 12)	HCV Testing Message Group (n = 22)	P-value
Sex	Female	42 (60.9%)	9 (75.0%)	16 (72.7%)	0.514
	Male	27 (39.1%)	3 (25.0%)	6 (27.3%)	
Race	Caucasian	48 (69.6%)	11 (91.7%)	14 (63.6%)	0.413
	African-American	14 (20.3%)	0 (0.0%)	5 (22.7%)	
	Other	7 (10.1%)	1 (8.3%)	3 (13.6%)	
Ethnicity*	Hispanic	8 (11.6%)	1 (8.3%)	1 (4.8%)	0.872
	Non-Hispanic	61 (88.4%)	11 (91.7%)	20 (95.2%)	
Physician Seen for Appointment	1	7 (10.1%)	1 (8.3%)	6 (27.3%)	0.342
	2	8 (11.6%)	2 (16.7%)	1 (4.5%)	
	3	19 (27.5%)	4 (33.3%)	9 (40.9%)	
	4	19 (27.5%)	4 (33.3%)	4 (18.2%)	
	5	16 (23.2%)	1 (8.3%)	2 (9.1%)	
Previous HCV Test	Yes	21 (30.4%)	3 (25.0%)	4 (18.2%)	0.589
	No	48 (69.6%)	9 (75.0%)	18 (81.8%)	

Although not statistically significant, participants who received the HCV text message were 2.63 (95% CI: 0.98, 7.07) times more likely to have an HCV test ordered than patients in the background population (Table 2). Receiving the general health message was not associated with having an HCV test ordered (Table 2). Among participants who were sent the HCV testing message and had a test ordered on their appointment date, none had a previous HCV test recorded in their EMR.

**Table 2:** Hepatitis C Antibody Test Orders among 1945-1965 Birth Cohort Patients HCV: Hepatitis C virus

	Background Population	General Health Message Group	HCV Testing Message Group
Number of Patients	69	12	22
HCV Test Orders on Appointment Date, n (%)	19 (27.5%)	5 (41.7%)	11 (50%)
Odds Ratio (95% Confidence Interval)	Reference	1.88 (0.53, 6.65)	2.63 (0.98, 7.07)

## Discussion

Our proof of concept study demonstrated the feasibility of using a patient-directed text message campaign to promote HCV testing in primary care. We found a trend toward increased HCV test orders among patients who received an HCV-related text message compared to those who received no text message. This suggests a personalized and timely HCV text message that educates and activates patients could improve HCV testing. By providing patients with a cue to action one hour before their appointment, our text may have prompted patients to set their own agendas for the appointment.

Our study had limitations. The small size of this proof of concept study limits generalizability. Enrolled participants may have been more engaged in their healthcare and therefore more likely to ask about HCV testing. To obtain physician buy-in, they were provided a brief overview of the study, which may have influenced physician behavior. However, in an attempt to reduce this study's influence on HCV test order practices of physicians, they were not involved in patient recruitment and were not made aware of which patients had chosen to participate.

Although studies have shown encouraging results using physician-based HCV testing interventions, such as EMR reminders and physician education, HCV testing remains suboptimal [4,6]. Thomson et al. found that 71% of primary care physicians thought increased patient education would help improve HCV testing [4]. In addition, Levine et al. found that HCV text messages would be acceptable to patients [7]. Therefore, a patient-based educational prompt, such as our HCV text message campaign, could complement physician-based interventions to increase testing rates.

## Conclusions

In summary, HCV testing rates remain low and novel methods are needed to improve testing rates. This proof of concept study demonstrated the feasibility of text messaging to promote HCV testing among birth cohort patients. Participants who were sent an HCV message were more likely to have an HCV test ordered compared to participants who were sent a general health message or patients who were not sent a message. Although these differences were not statistically significant due to the small sample size of this study. Further research is needed to test the efficacy of our campaign in a larger, more diverse patient population.
